# Unstable environment of coastal lagoons drives genetic variation in the amphipod *Quadrivisio lutzi*

**DOI:** 10.1590/1678-4685-GMB-2023-0094

**Published:** 2023-10-13

**Authors:** Mariana Sampaio Xavier, Paulo Cesar Paiva, Laura Isabel Weber

**Affiliations:** 1 Universidade Federal do Rio de Janeiro, Instituto de Biologia, Programa de Pós-Graduação em Biodiversidade e Biologia Evolutiva, Rio de Janeiro, RJ, Brazil.; 2 Universidade Federal do Rio de Janeiro, Instituto de Biologia, Departamento de Zoologia, Rio de Janeiro, RJ, Brazil.; 3 Universidade Federal do Rio de Janeiro (UFRJ), Instituto de Biodiversidade e Sustentabilidade (NUPEM), Laboratório de Biologia Molecular, Macaé, RJ, Brazil.

**Keywords:** Crustacea, Maeridae, Brazil, population genetics, mtDNA

## Abstract

The freshwater/brackish amphipod *Quadrivisio lutzi* inhabits coastal lagoons, highly unstable environments subject to sudden inflow of marine water. Our aim was to evaluate how the genetic composition varies in these populations. Brazilian populations were compared by *16S rRNA* and *COI* gene sequences. The genetic structure of four Rio de Janeiro amphipod populations was evaluated during the period of 2011-2019 by *COI*. Rio de Janeiro population was compared with Alagoas and São Paulo populations, which was genetically distinct, at species level (*16S, d* > 7%; *COI*, *d* >14%). The genetic structure in Rio de Janeiro showed the Imboassica subpopulation as the most divergent (Imboassica & Carapebus, *F*_*ST*_ = 0.238), followed by Lagamar population (Lagamar & Carapebus, *F*_*ST*_ = 0.049). The geographic distance and urbanization around these lagoons explain the degree of genetic isolation of these amphipod subpopulations. Paulista and Carapebus populations were not structured. Temporal variation in haplotype number and frequency were evident in both populations that were evaluated (Carapebus and Imboassica). Changes in salinity and water volume variation at these lagoons may be responsible for the observed changes in genetic composition, which may be the results of genetic drift effects over temporally fluctuating size subpopulations, without loss of genetic diversity.

## Introduction

Complex coastal lagoon systems are observed along the Brazilian coast (Esteves, 1998). At the north of the State of Rio de Janeiro (RJ), a lacunar coastal system was formed in the Campos basin during the Holocene (~5,000 BPY) by sea transgression and regression events (Esteves, 2011a). Most of these lagoons are within the National Park Restinga do Jurubatiba (PARNA Jurubatiba). The PARNA Jurubatiba represents a diverse ecosystem of eighteen coastal lagoons with different physicochemical properties (Enrich-Prast et al., 2004; Silva and Molisani, 2019). Coastal lagoons are highly unstable environments due to local variations in precipitation, evaporation (Kjerfve, 1994) and the intrusion of marine water due to the frequent rupture of sand barriers, which challenges the survival of most local freshwater species (Esteves, 1998; Camara et al., 2018; Santi et al., 2020).

Salinity is an important environmental parameter for many invertebrate species; for example, it determined the spatial structure of mussels (Blot et al., 1989) and the distribution of stenohaline amphipods (Zaabar et al., 2015). The amphipod *Quadrivisio lutzi* (Shoemaker, 1933) inhabits some of the coastal lagoons of the north of the State of Rio de Janeiro, and within the PARNA Jurubatiba. This amphipod species shows persistent populations in Carapebus and Imboassica lagoons, which has been attributed to the high reproductive potential (Medeiros and Weber, 2016). Brazilian records of *Q. lutzi* include the north of the State of Alagoas (Schellemberg, 1938) and the state of São Paulo (Leite et al., 1980; Wakabara et al., 1991). The type locality of the species is Georgetown, British Guiana, where it was originally described in the genus *Pseudoceradocus* (Shoemaker, 1933). It has also been registered for the Gulf of Mexico and Venezuela (Escobar-Briones et al., 2002; Martín et al., 2002; Capelo et al., 2004; Ortíz et al., 2007); and for Aruba and Bonaire islands (Stephensen, 1933), at which localities it was described as *Q. occidentalis,* a synonym of *Q. lutzi*. All records so far of *Q. lutzi* are from coastal environments, from brackish estuarine to freshwater habitats (Stephensen, 1933; Leite *et al.*, 1980; Ortíz *et al.*, 2007).

Vertebrate and invertebrate species inhabiting coastal lagoons have been genetically studied, showing mostly high levels of haplotype diversity and endemism, which gives these ecosystems high ecological and genetic importance (Vergara-Chen et al., 2010a,b; Mejri et al., 2011; Vasileiadou et al., 2016; Seixas et al., 2018).

Changes in population abundance has been observed in the amphipod *Q. lutzi* after sudden changes in salinity. Although the amphipod population has been shown to recover in a few months, no genetic study is so far done on how its genetic composition is affected by unstable environments. Therefore, the aim of this study was to evaluate changes in genetic composition and diversity along time at different coastal lagoons situated at the north of the State of Rio de Janeiro, Brazil.

## Material and Methods

### Amphipod sampling

**Figure 1 -:**
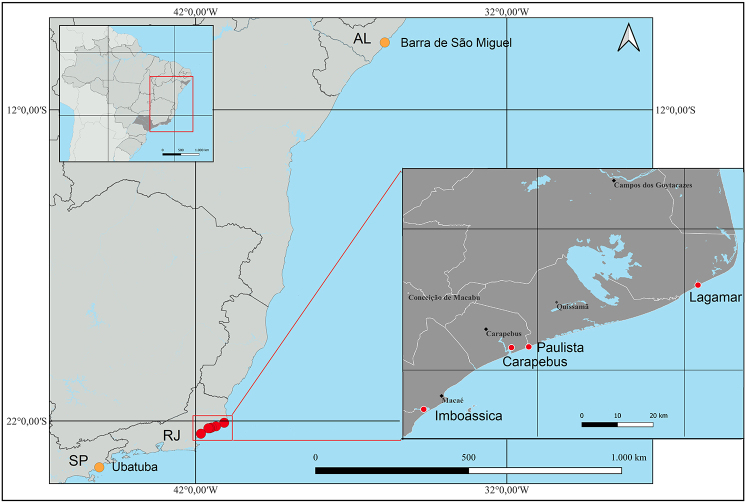
Sites where the amphipod *Quadrivisio lutzi* was collected.

*Quadrivisio lutzi* amphipods were collected by hand from macrophyte roots, from algae or under vegetal debris at shallow waters in four coastal lagoons in the state of Rio de Janeiro and in two river/lagoon outlets from the states of Alagoas and São Paulo (Figure 1). Coordinates and salinity were obtained at each location (Table 1). Amphipods were then fixed in 92.8% ethanol and stored in 1.5 mL microtubes.

**Table 1 -:** Sampled locations along the Brazilian coast of the amphipod *Quadrivisio lutzi*.

Locality	Coordinates	Collection date	Salinity (ppt)	Number of amphipods	
				*16S*	*COI*
**State of Alagoas**					
Roteiro lagoon, Barra de São Miguel	9º50’6”S; 35º55’19.2”W	August 27, 2021	0.2	1	4
		December 19, 2021	0.2	1	1
**State of Rio de Janeiro**					
Lagamar outlet, Campos dos Goytacazes	22º3’35.1”S; 41º5’00.7”W	April 24, 2019	6.7	7	28
Paulista lagoon, Quissamã*	22°14’2.5”S; 41º32’40.4”W	April 1, 2014	0.6	10	23
		August 8, 2015	0.5	4	5
Carapebus lagoon, Carapebus*	22°14’11.9”S; 41º35’28.8”W	October 21, 2011	0.3	0	39
		November 1, 2013	0.6	0	4
		November 1, 2014	8.3	6	1
		August 11, 2015	9.3	9	28
		April 13, 2016	3.9	0	7
		April 24, 2019	4.5	9	41
Imboassica lagoon, Macaé	22º24’42.3”S; 41º49’48.5”W	March 7, 2016	0.3	3	36
		July 2, 2018	0.5	3	19
**State of São Paulo**					
Escuro River, Ubatuba	23º29’27”S; 45º05’50”W	December 19, 2020	2.1	2	0
		February 12, 2022	0.6-3.4	2	0

### DNA extraction, amplification, and sequencing

Whole amphipods were homogenized individually with sterilized glass sticks‚ and then DNA extraction was performed using Phenol/Chloroform/Proteinase-K (Sambrook et al., 1989) or Chelex-100 (Sigma) protocols (Hoelzel, 1998) with modifications. For Chelex extraction, each amphipod was homogenized in 75 µL lysis buffer (0.2 mM Tris-HCl, 0.02 mMEDTA, pH 8.0). Then‚ 75 µL Chelex 12% solution and 30 µL Proteinase K (10 mg/mL) were added, mixed with a vortex mixer‚ and incubated overnight at 55 ºC.

Amplifications of the cytochrome *c* oxidase, subunit I (*COI*), and 16S rRNA (*16S*) mitochondrial genes were performed by polymerase chain reaction (PCR) using universal primers and primers designed specifically for *Q. lutzi* (Table 2). PCR reactions (25 µL) were performed with 1-10 µL of extracted DNA or dilutions in double distilled water (1:2, 1:5, 1:10, 1:30, 1:50, 1:100); 1x Buffer; 3 mM MgCl_2_; 0.12% Triton-X-100; 0.24 mM dNTPs mix; 0.4 mM of each primer; 2 U of GoTaq® DNA polymerase (Promega, Madison, WI, USA). PCR reactions were submitted in a Mastercycler gradient thermocycler (Eppendorf, Hamburg, Germany) to the following cycles: 1 cycle at 94 ºC for 4 min; 36 cycles for 1 min at each of the following temperatures: 94 ºC, 48 ºC -59 ºC (*COI*) and 52 ºC-57 ºC (*16S*) and 72 ºC; and one final cycle at 72 ºC for 10 min. All PCR products were purified and sequenced by Macrogen Inc., Korea, using the automated Sanger dideoxide method.

**Table 2 -:** Primers used for the amplification by PCR of mitochondrial genes (*COI* and *16S*) of the amphipod *Quadrivisio lutzi*.

Target gene	Primer sequence (5’-3’)	Expected size (bp)	Reference
*COI*	LCO1490: TAAACTTCAGGGTGACCAAAAAATCA	710	Folmer et al. (1994)
	HCO2198: GGTCAACAAATCATAAAGATATTGG		
	QCOI-R1: TAGGTGCTGGAATAAAATAGGG	685	Weber, L.I. unpublished
	QCOI-F1: ACACTCTACCTTATTACCGGAT	655	
	QCOI-R1: TAGGTGCTGGAATAAAATAGGG		
	QCOI-F3: CGNATAGARCTTTTAGTCCC	485	Present study
	QCOI-R3: AGRGAGAGTAGAAGAAGTGT		
	QCOI-F4: TGRGCAGGACTYCTRGGTAGATC	545	
	QCOI-R5: ATRGCCCCTGCTAAKACRGG		
*16S*	16sar: CGCCTGTTTATCAAAAACAT	515	Palumbi et al. (2002)
	16sbr: CCGGTCTGAACTCAGATCACGT		
	Q16s-F2: CGTACATAGTACCTGCCCAGTG	445	Present study
	Q16s-R3: GGATGAACAATCCCACTCTC		

*Situated within the PARNA Jurubatiba.

### Data analysis

Sequences were edited with ChromasPro (McCarty, 1998) and Geneious Prime software (Geneious 11.0.14.1, 2022). Alignments were done using CLUSTAL W (Higgins et al., 1994) implemented in MEGA11 software (Tamura et al., 2021). Translation of *COI* sequences was done by aligning with *Daphnia pulex* (Accession No. NC000844) and *Parhyale hawaensis* (Accession No. NC039402) *COI* gene, using the Invertebrate Mitochondrial Code, for identifying the position of the amplified fragment in the gene and to recognize synonymous and non-synonymous mutations. Sequences obtained for Rio de Janeiro populations were submitted to the Nucleotide GenBank database (*16S,* OQ361834-OQ361842; *COI,* OQ401341-OQ401385).

The genetic divergence between Rio de Janeiro population and the amphipod populations from the states of Alagoas and São Paulo were obtained by Kimura 2-parameter model (d; Kimura, 1980) for the *16S* and *COI* genes, using MEGA 11 software. Trees were constructed based on maximum likelihood (ML) and Bayesian inference (BI) at the *16S*, *COI* and concatenated data sets, using evolutionary models determined by jModelTest 2.1 (Darriba et al., 2012) under the Akaike criterium (GTR+G model and HKY+I+G, respectively). Three outgroups were included in the analysis for tree rooting: for *16S, Elasmopus nkjaf* (Accession No. LC215808, LC215809), Maeridae; *Quadrimaera pacifica* (Accession No. AB432980), Maeridae; and *Gammarus pulex* (Accession No. AJ269626), Gammaridae. For *COI*: *E. nkjaf* (Accession No. LC215812, LC215813); *Melita nitida* (Accession No. MH826277, MH826279), Melitidae; and *G. pulex* (Accession No. MN400977). *COI* trees were performed only for Alagoas and Rio de Janeiro populations, because it was not possible to obtain more amphipods from Ubatuba, São Paulo, although sampling efforts were made. A heuristic search of the ML tree was performed using Garli 2 software (Zwickl, 2006) with 1,000 replicates and 1,000 bootstrap resampling for tree branch support. The BI analysis was performed using Markov chain Monte Carlo algorithms with four simultaneous chains for 10,000,000 generations with standard deviation of Split frequencies is below 0.01 using MrBayes 3.2 software (Ronquist et al., 2012) and the optimization criterion of the maximum posterior probability. The quality of the Bayesian sampling was evaluated by Tracer v1.7.1 software (Rambaut et al., 2018) using the burn-in value applied with MrBayes to obtain the mean posterior probability of the consensus tree and the ESS values. Branch support of the BI tree was represented by the posterior probability of the clades obtained using MrBayes software. Broad estimations of times since divergence between pairs of lineages were calculated using the conventional rate of mitochondrial nucleotide substitution of 2 % per mya, using t= 1/2d/µ (Brown et al., 1979).

The genetic structure and temporal variation of the amphipod population of the north of the state of Rio de Janeiro (Rio de Janeiro population) was evaluated using *COI* gene. A TCS network (Clement et al., 2002) was performed using PopArt software (Leigh and Bryant, 2015) for amphipod haplotypes from four lagoons (Lagamar, Paulista, Carapebus and Imboassica; Figure 1), showing haplotype frequencies. Nucleotide diversity and the pairwise population structure parameter, *F*_*ST*_ , were obtained by Arlequin (Excoffier and Lischer, 2010). The genetic divergence of the populations was evaluated by the Kimura 2-parameter model using MEGA 11 software. Haplotype diversity and the neutrality tests of Tajima (1989) and Fu (1997) were obtained using DnaSP v6 software (Rozas et al., 2017) for each population/year compared. Genetic changes through time were evaluated for the two largest amphipod populations (Carapebus and Imboassica) with evidence of bar opening events and strong salinity changes.

Rainfall data were obtained from Instituto Nacional de Meteorologia (INMET) from the daily registrations of the automatic station A608 at Macaé, state of Rio de Janeiro, Brazil. The sum of rainfall at each month between 2011 and 2019 was calculated.

## Results

Seven haplotypes for *16S* were obtained from 48 sequences with a length of 425 bp. For *COI*, 22 haplotypes of 236 sequences with a length of 588 bp were obtained.

### Divergence of amphipod populations along the Brazilian coast

**Figure 2 -:**
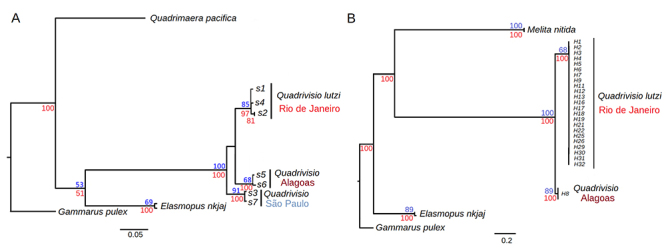
Brazilian populations of *Quadrivisio*. Bayesian inference trees. A) based on *16S* sequences, showing the divergence between Rio de Janeiro, Alagoas and São Paulo populations. B) based on *COI* sequences, showing the divergence between Rio de Janeiro and Alagoas populations. Numbers (in blue) bootstrap branch support; (in red) posterior probability from Bayesian inference.

The degrees of divergence among the Brazilian populations of *Q. lutzi* are shown in Figure 2. Populations at different states show independent branches with high bootstrap support and Bayesian posterior probability (Figure 2a). Genetic distances among them, confirm that Rio de Janeiro population is highly divergent from Alagoas (16S, *d* = 0.0795 ± 0.0003) and to São Paulo (16S, *d* = 0.0879 ± 0.0007) populations. Alagoas and São Paulo were the most divergent (16S, *d* = 0.0969) populations. The high divergence observed between Rio de Janeiro and Alagoas population was confirmed with *COI* gene sequences (Figure 2b) which showed high distance (*d* = 0.1472).

###  Population structure of the amphipod *Q. lutzi* at the north of the State of Rio de Janeiro (Rio de Janeiro population) 

Four coastal lagoons were found with large numbers of amphipods (Lagamar, Paulista, Carapebus and Imboassica). Other lagoons from which a few amphipods were collected in previous sampling events, but in which they were no longer found (Maria Menina, Ubatuba, Preta and Garças), were not included in the analysis. Pairwise genetic distance, *F*_ST_ and diversity parameters for the four studied populations are shown in Table 3. The mean genetic distance among the four populations was *d* = 0.0009 ± 0.0002. Imboassica showed a significantly high *F*_ST_ from all other populations (0.163-0.238), showing that Rio de Janeiro population is structured. Paulista amphipod population did not show significant differences from Lagamar and Carapebus; and Lagamar showed significant, but low level of structuring with Carapebus (Table 3).

**Figure 3 -:**
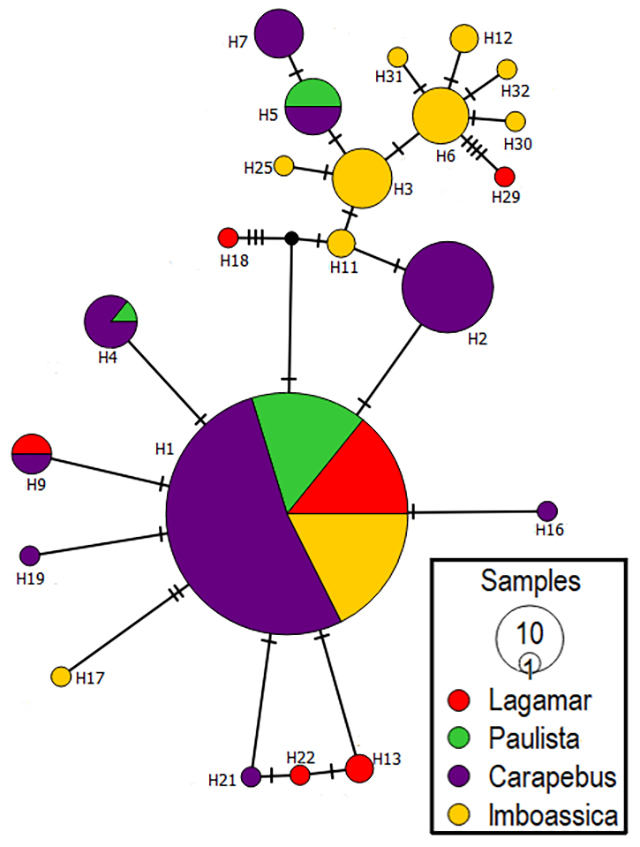
Rio de Janeiro population of *Quadrivisio lutzi*. Network of *COI* haplotypes found in different lagoons. Haplotype frequencies are relative to circle size.

A total of 22 haplotypes with a total of 24 segregating sites of which 10 were parsimony informative, were found at Rio de Janeiro population. The most frequent haplotype (H1) was represented at all subpopulations (Figure 3). Each subpopulation (Lagamar, Carapebus-Paulista and Imboassica) had haplotypes found nowhere else. All diversity parameters showed Imboassica as the most diverse subpopulation, followed by Carapebus. (Table 3). Time since divergence of Imboassica subpopulation was estimated at 86,000 years ago and divergence between Lagamar and Carapebus was estimated around 50,000 years ago.

**Table 3 -:** Genetic structure of Rio de Janeiro population of *Quadrivisio lutzi*. Pairwise Kimura 2-parameter distance (above the diagonal), *F*_*ST*_ values (below the diagonal) and diversity parameters of amphipod populations from different lagoons, based on *COI* sequence analysis. Significant values (*p* < 0.05) are shown in bold.

Population (N)	Lagamar	Paulista	Carapebus	Imboassica	Diversity Parameters		
					NH	HD	ND
Lagamar (28)	***	0.00207	0.00198	0.00394	6	0.439	0.212 ± 0.154
Paulista* (28)	0.036	***	0.00175	0.00345	3	0.315	0.185 ± 0.140
Carapebus* (108)	**0.049**	0.000	***	0.00343	9	0.545	0.166 ± 0.126
Imboassica* (55)	**0.231**	**0.163**	**0.238**	***	10	0.705	0.376 ± 0.234

*Include all years of collections. (NH) Number of haplotypes found at each lagoon; (HD) haplotype diversity; (ND) nucleotype diversity.

###  Genetic changes along time in the Rio de Janeiro population of *Q. lutzi*

Temporal changes were observed in Carapebus and Imboassica subpopulations (Table 4). The population structure parameter, *F*_ST_, showed that Imboassica amphipod subpopulation increased its divergence from Lagamar and Carapebus-Paulista subpopulations from 2016 to 2018; and Carapebus diverged significantly from Lagamar and Paulista in 2019, while no such differences were show previous years (2011 and 2015; Table 4).

**Table 4 -:** Temporal genetic changes in Rio de Janeiro population of *Quadrivisio lutzi*. Pairwise Kimura 2-parameter distance (above the diagonal), *F*_*ST*_ values (below the diagonal) and diversity parameters of amphipod populations from different lagoons and years, based on *COI* sequence analysis. Significant values (*p* < 0.05) are shown in bold. In yellow, comparisons from the same or following year.

Population and Year (N)	Lagamar 2019	Paulista 2014/15	Carapebus 2011	Carapebus 2015	Carapebus 2019	Imboassica 2016	Imboassica 2018	Diversity parameters		
								NH	HD	ND
Lagamar 2019 (28)	***	0.002	0.002	0.002	0.003	0.003	0.020	6	0.439	0.2115± 0.1536
Paulista 2014/15 (28)	0.036	***	0.001	0.001	0.002	0.003	0.019	3	0.315	0.1849± 0.1395
Carapebus 2011 (39)	0.027	0.014	***	0.001	0.002	0.003	0.019	2	0.157	0.0991 ± 0.0907
Carapebus 2015 (28)	0.009	0.024	0.000	***	0.002	0.003	0.020	4	0.221	0.097 ± 0.0904
Carapebus 2019 (41)	**0.190**	**0.114**	**0.250**	**0.220**	***	0.003	0.019	6	0.645	0.2024 ± 0.1471
Imboassica 2016 (36)	**0.160**	**0.102**	**0.208**	**0.198**	**0.120**	***	0.019	7	0.571	0.3466 ± 0.2215
Imboassica 2018 (19)	**0.474**	**0.418**	**0.583**	**0.566**	**0.361**	**0.150**	***	7	0.792	0.3543± 0.2339

(NH) Number of haplotypes found at each lagoon; (HD) haplotype diversity and (ND) nucleotype diversity.

**Figure 4 -:**
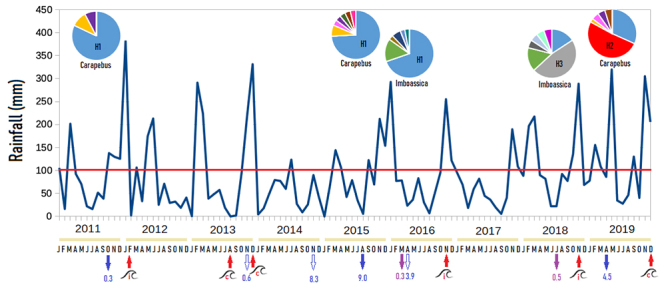
Changes in *COI* haplotype frequencies (circles) between 2011 and 2019 in the amphipod *Quadrivisio lutzi* at two localities (Carapebus and Imboassica lagoons) are shown over rainfall variation (INMET). Most common haplotypes (H1, H2 and H3) are indicated within the circle and other haplotypes are represented by different colors. Sandbar breaks are represented by red arrows at the localities of Carapebus (c) and Imboassica (i); and blue and purple arrows indicate sampling events at Carapebus and Imboassica, respectively. The number under sampling events indicates the salinity at the time of collection.

Diversity parameters (haplotype and nucleotype diversity) also changed during time in Carapebus and Imboassica subpopulations, increasing in 2018/2019 compared to 2015/2016 (Table 4). In more recent years (2018/2019) a dramatic change was observed in the most common allele (H1) from 2011-2016, turning H2 and H3, the most common alleles in Carapebus and Imboassica, respectively. In 2015, the Carapebus amphipod population showed an increase of low frequency haplotypes compared to 2011; and in 2019, low-frequency haplotypes declined (Figure 4; Table 5). In Imboassica, low-frequency haplotypes of 2016 increased their frequencies in 2018 (Figure 4; Table 5). The neutrality tests were non-significant for most populations at the different years; only Lagamar (Tajima’s *D* = -2.1039, *p* < 0.05; Fu’s *Fs* = -1.097, *p* < 0.05) and Carapebus subpopulation of the year 2015 (Tajima’s *D* = -2.2295, *p* < 0.01; and Fu’s *Fs* = -3.562, *p* < 0.02) showed deviation from neutrality.

**Table 5 -:** Temporal changes in haplotype frequency in populations of *Quadrivisio lutzi* from Carapebus and Imboassica lagoons.

Haplotypes	Population and Year (N)				
	Carapebus lagoon			Imboassica lagoon	
	2011 (39)	2015 (28)	2019 (41)	2016 (36)	2018 (19)
H1	0.820	0.741	0.317	0.697	0.157
H2	0	0	0.512	0	0
H3	0	0	0	0	0.474
H4	0.103	0.074	0.024	0	0
H5	0	0.037	0.049	0	0
H6	0	0	0	0.152	0.157
H7	0.077	0.037	0.049	0	0
H9	0	0	0.049	0	0
H11	0	0	0	0.030	0.053
H12	0	0	0	0.061	0
H15	0	0.037	0	0	0
H16	0	0.037	0	0	0
H17	0	0	0	0	0.053
H19	0	0.037	0	0	0
H25	0	0	0	0	0.053
H30	0	0	0	0.030	0
H31	0	0	0	0.030	0
H32	0	0	0	0	0.053

## Discussion

### Divergence of amphipod populations along the Brazilian coast

Along the Brazilian coast, three distinct populations (Alagoas, Rio de Janeiro and São Paulo) with high levels of divergence, a strong indication of the presence of more than one species in Brazil for the genus *Quadrivisio*. The levels of divergence (*16S*, > 7 %; *COI, >* 14%) found between them are higher than those found at *16S* locus for conspecific crustacean populations (in crabs, 1.3%, Avise et al., 1994; copepods, 0.3-2.6%, Bucklin et al., 1995; amphipods: 1-3.9%, Jażdżewska and Mamos, 2019). Interspecific distances at *16S* locus have been reported for crustaceans within the range of 4.4 to 25.7% (Machado et al., 1993; Bucklin *et al.*, 1995; France and Kocher, 1996). The high level of divergence at *COI* gene found between Alagoas and Rio de Janeiro population (> 14%) also support the multispecific status of the genus *Quadrivisio*. *COI* gene has great potential to complement traditional taxonomy in the identification of crustacean species (Costa et al., 2007). In accordance to Costa *et al.* (2009), studying 15 species of the genus *Gammarus* and three pair of species of other amphipod genera, intraspecific range of distances was 0-4.3%, while the interspecific range was 5.2-34.2%. Corroborating that the degree of divergence of the Brazilian populations of *Quadrivisio* from different states is within the range of interspecific populations, it is a strong indicative of the presence of cryptic or semi-cryptic species of this genus in the surveyed area. The morphological description and identification of diagnostic characters will be necessary for the delimitation and recognition of these potential species.

### Genetic structure of the Rio de Janeiro amphipod subpopulations

Taxonomic reviews and catalogs of Brazilian Amphipoda have already shown the sparse and rare distribution of *Quadrivisio* (Wakabara et al., 1991; Serejo and Siqueira, 2018). Sampling efforts in coastal lagoons of the states of Espírito Santo, Santa Catarina and Rio Grande do Sul have not reported the species (unpublished). Despite the sampling effort of the present study in the known locations of the *Quadrivisio* distribution in Brazil, the abundance was very low in environments permanently open to the sea. The low representation of the species may be reflecting historical events on its distribution and environmental requirements of the species.

The population of *Q. lutzi* in the state of Rio de Janeiro is abundant and it was found to be highly structured, as expected from fragmented environments (Astolfi et al., 2005). The amphipod population is divided into three subpopulations (Lagamar, Carapebus-Paulista and Imboassica). Levels of differentiation among them may be explained by the degree of isolation due to the geographic distance that separate them and by the progressive urbanization around them, in the cases of Imboassica and Lagamar. Connectivity in the past may have moderated differentiation between them, in the cases of Lagamar and Carapebus-Paulista subpopulations; and present day connectivity may prevent further differentiation between amphipods from different lagoons, in the cases of Carapebus and Paulista.

In the past, a large floodable area, called the “Pantanal Fluminense”, interconnected Lagoa Feia to all the PARNA Jurubatiba lagoons (Lamego, 1946), which includes Paulista and Carapebus. Lagamar lagoon is a remnant of the Lagoa Feia drainage canal (Soffiati, 2013), that became isolated from the PARNA Jurubatiba with the progression of drainage activities and urbanization (Silva and Molisani, 2019). Past connectivity may explain the present low values of subdivision between Lagamar and Carapebus-Paulista subpopulations. Although genetic differences were lower in previous years between Lagamar and Carapebus-Paulista subpopulations, the increased urbanization around Lagamar will prevent any future gene flow between them, therefore it is expected that genetic differences will increase with time.

Paulista and Carapebus lagoons show variable connectivity, determined by an inner arm of Carapebus lagoon, which may increase its extent in rainy periods allowing gene flow (Esteves, 2011b) or became interrupted on severe dry seasons.

Imboassica was the most genetically differentiated subpopulation. According to Esteves (2011a), the Imboassica River micro basin was formed by sea transgression and regression events during the Holocene (~5,000 years ago). At the time, the river flow was small and the sand deposition by winds and currents led to the formation of the Imboassica lagoon orthogonal to the coastline (Silva and Molisani, 2019). About 3,000 years ago‚ the first sandbar was formed (paleobar), semi-isolating the lagoon from the sea. A probable rupture of the paleobar happened 1,000 years ago, advancing the lagoon to its current position (Panosso et al., 1998). Imboassica is situated at ~29 km from Carapebus lagoon. Although Cabiúnas lagoon is closer to Imboassica (~18 km) than Carapebus lagoon, physicochemical conditions at Cabiúnas and Comprida lagoons are not suitable for amphipod survival. Imboassica lagoon has also been affected by urbanization and farming, decreasing its extent, and causing urban waste contamination at some points (Barreto, 2009).

The genetic divergence of Imboassica from the other Rio de Janeiro subpopulations suggests that divergence may have started around 86.000 years ago, dating back to the beginning of the fourth transgressive-regressive cycle at the Atlantic South American coast (Carreño et al., 1999). This estimation is much older than suggested by Esteves (2011a), of ~5,000 years ago of the Imboassica lagoon emergence. Repetitive drastic changes in lagoon water volumes and salinities may have increased divergence among subpopulations submitted to different regimens of stochastic and directional selective events. Therefore, in populations submitted to unstable environments with temporal variation of effective population size, any estimation of date from divergence should be interpreted carefully (Whitlock, 1992; Pisa et al., 2019). Nevertheless, genetic divergence among Rio de Janeiro subpopulations suggests that amphipod colonization in the region occurred before the formation of the contemporary lagoons.

The long-term isolation of Imboassica lagoon may explains the presence of exclusive haplotypes, which is characteristic of coastal lagoons (Pérez-Ruzafa et al., 2019). Micro-invertebrates transport at different life cycle stages may occur by waterbirds (Silva et al., 2021), but may not be frequent, having minimal effect on gene flow among large isolated amphipod populations.

### Temporal genetic variation in Carapebus and Imboassica subpopulations

Strong variation in genetic composition was observed at both localities (Carapebus and Imboassica) in the years of 2018/2019 compared to previous years. The genetic changes were evident on the increased diversity of haplotypes and the change of the most common haplotype at each subpopulation. Deviation from neutrality indicates population expansion at Carapebus in 2015‚ which predict large population size. Population growth determine the increase of amphipods with rare haplotypes, therefore retaining diversity (Pavesi and Matthaeis, 2009; Vergara-Chen et al., 2010b; Pavesi *et al.*, 2011). At Carapebus lagoon, optimal environmental conditions were observed (Salinity of 0.3-0.6 ppt; large rain volumes) from 2011 until middle of 2013 (Figure 4), when large volumes of amphipods were easily obtained. Large reproductive potential (Medeiros and Weber, 2016) may have contributed to population increase at these years. In late 2013 the sandbar was artificially opened twice. Although there was a drastic salinity increase after the sandbar breaks in 2013, amphipod population appears to have been unaffected. In March of 2014, amphipods were not easily found close to the sandbar of Carapebus lagoon, where salinity was > 13 ppt; however, amphipods were found in the innermost part of Carapebus lagoon and in Paulista lagoon, where water remained at 0.5-0.6 ppt of salinity. The artificial sandbar opening in the end of 2013 was followed by a severe dry period that lasted from the beginning of 2014 to the end of 2015 (Figure 4). Although, no genetic differentiation were found at Carapebus in August of 2015, when amphipods were abundant at 9.3 ppt of salinity. Therefore, the dry period did not affect the amphipod population. What happened in the Carapebus amphipod population between August 2015 and April 2019 is discussed below.

The Imboassica lagoon also showed a drastic change in genetic composition from March 2016 to July 2018. Imboassica lagoon suffered a strong drop of water level and a sudden increase of salinity in November 2016. Imboassica is smaller (3.3 km^2^) than Carapebus lagoon (6.5 km^2^; Panosso et al., 1998) and it is surrounded by urbanized areas that motivate frequent artificial sand bar openings to prevent the flooding of houses around the lagoon. In addition, since 1980 the Imboassica lagoon gradually deteriorated, reaching in 2015 the hypertrophic condition (Silva and Molisani, 2019). Therefore, amphipod population is restricted to the southern anterior margin of the Imboassica lagoon, without routes or other areas to escape under conditions of salinity changes.

In both lagoons, the change of the most frequent haplotype may have happened by a drastic temporarily reduction in population size, followed by a sweepstakes chance event that led to the increase in frequency of new dominant haplotypes mainly due to the effect of genetic drift. At Imboassica, certainly the sudden lagoon volume reduction and salinity increase may explain the severe reduction in population size. However, for Carapebus subpopulation, may not be the case. The environmental instability caused by the sudden intrusion of seawater in Carapebus and Imboassica lagoons has driven changes in fish assemblage (Camara et al., 2018). Euryhaline amphipod predators that deal well with salinity variations may have increased their population size intensifying amphipod predation and therefore reducing their population size. On the other hand, osmoregulation of the amphipod *Q. lutzi* suggest that osmotic stress may be related to population decline in Carapebus (unpublished data). During 2014 and most of 2015, surviving and newborn amphipods had to live in areas close to the sea under a salinity range of 8-13 ppt, on which they are able to osmoconform (unpublished data). At the end of 2015, salinity dropped, and amphipods needed to activate the hyper-regulation system, which would have demanded time and energy, causing probably population size reduction in the amphipod at Carapebus lagoon.

We do not understand exactly how and when different mechanisms of osmoregulation are activated in the new born amphipods or in adult amphipods, which remained most of their life in a specific level of salinity. Therefore, we cannot rule out completely the possibility that selection may have taken place when population size was still elevated, acting against amphipods not well adapted to a specific new salinity regimen.

The instability in coastal lagoons due to strong water volume and salinity variations has driven changes in the genetic composition of *Q. lutzi* by genetic drift acting over a fluctuating population size, which causes changes in haplotype frequencies, without diversity loss.

The high diversity and endemism observed in coastal lagoons (Vergara-Chen et al., 2010b; Milana et al., 2012; Pérez-Ruzafa et al., 2019) and the ability of species to survive in such unstable environmental conditions (González-Wangüemert et al., 2006; Pérez-Ruzafa *et al.*, 2019), reinforce the need of protection of these peculiar ecosystems.
